# Exposure to e-cigarette advertising, attitudes, and use susceptibility in adolescents who had never used e-cigarettes or cigarettes

**DOI:** 10.1186/s12889-020-09422-w

**Published:** 2020-09-04

**Authors:** Lijun Wang, Jianjiu Chen, Sai Yin Ho, Lok Tung Leung, Man Ping Wang, Tai Hing Lam

**Affiliations:** 1 School of Public Health, University of Hong Kong, 7 Sassoon Road, Pokfulam, Hong Kong, P. R. China; 2 School of Nursing, University of Hong Kong, 21 Sassoon Road, Pokfulam, Hong Kong, P. R. China

**Keywords:** E-cigarettes, Advertising, Susceptibility, Attitudes, Adolescents

## Abstract

**Background:**

Tobacco advertisements have been banned in Hong Kong, but low intensity e-cigarette (EC) advertising can still be found in various media outlets. We investigated the associations between exposure to EC advertising and susceptibility to EC use in adolescents who had never used cigarettes or ECs, with potential mediation by attitudes towards ECs and the tobacco industry.

**Methods:**

The School-based Smoking Survey 2016/17 assessed exposure to EC advertising in the past 30 days, attitudes towards ECs and the tobacco industry, susceptibility to EC use, and other covariates. Generalised linear mixed models and multiple mediation analysis methods were used in data analyses.

**Results:**

Among 7082 students (mean age 14.9 years), 28.8% reported exposure to EC advertising. Exposure was associated with being uncertain about the harm of EC use, being tolerant towards ECs, believing the tobacco industry is respectable, and being susceptible to EC use; the magnitudes of these associations increased with the number of types of advertising exposed (0, 1, and ≥ 2) (all *p*-values < 0.01). These attitudinal factors were in turn associated with EC use susceptibility (all p-values < 0.001). The perception that tobacco companies make youth smoke was not associated with the advertising exposure or EC use susceptibility. Of the total effects of EC advertising on use susceptibility, only 16.6% were mediated by attitudinal factors: perceived harm (3.1%), attitudes towards ECs (8.2%), and attitudes towards the tobacco industry (4.9%).

**Conclusions:**

Even exposure to low intensity EC advertising was associated with susceptibility to EC use in adolescents who had never used cigarettes or ECs. The association was weakly mediated by tolerant attitudes towards ECs and the tobacco industry. The government should reinforce the regulations on EC advertising to protect adolescent health.

## Background

Tobacco use is a leading cause of preventable diseases and mortality. E-cigarettes (ECs) are claimed to be less harmful than combustible cigarettes [1] and to help cigarette smokers quit [2]. However, mounting evidence has shown their links with severe acute lung injuries, chronic respiratory and cardiovascular diseases, and deaths [3–8].

While the tobacco industry promoted ECs as safer alternatives for adults, they have become more popular than conventional tobacco products among youth. In fact, EC advertising targets minors as young as 12-year-olds, [9] and exposure to EC advertisements on television increased by 256% from 2011 to 2013 in US adolescents [10]. In recent years, ECs are increasingly promoted in social media targeting youth, such as Facebook, Twitter, Instagram and YouTube [11–13]. With its intensive and wide-reaching marketing in new media, JUUL has become the largest EC brand in less than 3 years [11]. Lacking clear and stringent regulations on EC advertising, many online stores and social media channels have no age warnings or age gating to exclude minors [14]. In US high school students, less than half (43.2%) perceived that EC use was harmful in 2017 [15]; current EC use increased by 135% from 11.7% in 2017 to 27.5% in 2019 [16, 17].

Studies on ECs in developing countries focused mainly on the prevalence of use and awareness. Ever EC use increased from 1.8 to 7.1% in never smoking adolescents over 17 months in 2014–15 in Argentina, and 6.5% of Mexican adolescents had ever used ECs in 2016 [18, 19]. China had one of the highest smoking rates in the world (52.1% current smoking in males in 2015), [20] but the prevalence of EC use was relatively low, with 3.1% adults in 2015 and 5.2% adolescents in 2017 being ever users [20, 21]. EC awareness in urban adult smokers increased from 29% in 2009 to 60% in 2015, and 70.6% middle and high school students were aware of ECs in 2017 in China [22, 23]. Hong Kong is one of the most developed regions in China, and its smoking rates remained low in the past decade (10.2% daily smoking in persons aged 15 and over in 2019) [24]. Stringent tobacco control measures are in place in Hong Kong, and all EC products containing nicotine are effectively banned. However, ECs with or without nicotine, including JUUL and ammo, are still available in online and local stores [25, 26]. The Smoking (Public Health) Ordinance (Cap 371) in Hong Kong has banned the sale of cigarettes and cigars to minors, but the ban does not include ECs.

Cohort studies have reported that adolescents exposed to online tobacco marketing were more likely to initiate tobacco use and progress to poly-tobacco use [27]; those who recalled EC advertisements at baseline were 77% more likely to be susceptible to EC use (adjusted odds ratio [AOR] = 1.77, 95% confidence interval [CI] 1.20–2.61) and twice more likely to use ECs (2.99, 1.50–5.97) within 6 months [28]. A randomised controlled trial (RCT) showed that exposure to EC advertising increased curiosity to try ECs (2.85, 1.07–7.61) in young adults who had never used cigarettes or ECs [29]. Also, the exposure was associated with receptivity to and positive attitudes towards ECs, and was inversely associated with perceived health risks of EC use [30, 31]. Qualitative studies reported that youth exposed to EC advertising regarded ECs as attractive and safer than combustible cigarettes, and described EC use as “fun”, “fitting in” and “looking cool” [32, 33].

The tobacco industry evaluated the effects of advertising campaigns based on the hierarchy-of-effects model [34]. It showed a causal linkage of cognition-affect-conation: Advertising exposure made people aware of the product and develop a feeling for it, which in turn influenced preference and consumption behaviours [35]. The receptivity to advertising varied by individuals, and adolescents with higher receptivity to cigarette advertising were more likely to become experimenters and established smokers [36]. Exposure to EC advertising was also linked to higher use susceptibility in young adults, partially mediated through perceived health benefits or harm reduction [37]. However, such association and mediation effects are still unclear in adolescents, who are less health conscious and more curious than adults. Besides, the World Health Organisation recommends exposing the tobacco industry’s deceptive and manipulative tactics as a tobacco control strategy, [38] and we found that knowing these tactics was associated with lower susceptibility to cigarette smoking in adolescents [39]. Therefore, we also included adolescents’ perceptions of the tobacco industry to examine its mediation effect in the association between exposure to EC advertising and susceptibility to EC use.

Hong Kong has banned all forms of tobacco advertising, promotion and sponsorships, but the ban does not explicitly include ECs or other new tobacco products. Large-scale marketing campaigns of ECs are absent, but low intensity EC promotions can still be found in both local stores and online media. We examined the associations between exposure to EC advertising and adolescent susceptibility to EC use through the mediation of attitudes towards EC and the tobacco industry in never users of cigarettes and ECs, in order to help establish the pathways and inform policies that counter the influence of EC advertising on adolescents. We hypothesised that (1) exposure to EC advertising is associated with tolerant attitudes towards ECs and the tobacco industry; (2) exposure to EC advertising is associated with being susceptible to EC use; and (3) the association between exposure to EC advertising and susceptibility to EC use is mediated by tolerant attitudes towards ECs and the tobacco industry.

## Methods

### Study design and sample

The School-based Smoking Survey 2016–17 included 8828 students in 36 schools from all 18 districts of Hong Kong. The schools were selected using stratified random sampling in proportion to the total numbers of schools in each district. All students (Secondary 1–6, equivalent to US Grade 7–12) in the selected schools were invited to complete an anonymous questionnaire. Parental consent was sought before the survey, and declining parents were to ask their children to return a blank answer sheet during the survey. Before the survey, class teachers explained to students that participation was voluntary and they could decline even with parental consent. Ethics approval for the study including parental consent procedure was granted by the Institutional Review Board of the University of Hong Kong/Hospital Authority Hong Kong West Cluster. The school- and student-level response rates were 18% and 92%, respectively. Non-participation of schools was usually due to administrative reasons, e.g. tight schedules, rather than smoking-related issues. More detailed survey methods have been described elsewhere [40]. In the present study, we only included students who had never used cigarettes or ECs because tobacco use is known to be associated with both exposure to EC advertising and susceptibility to EC use, which could lead to confounding bias and exaggerate the associations in ever cigarette/EC users.

### Measurements

Cigarette smoking status was assessed by “Please read the following sentences and choose the one that suits you most” with options: “I have never smoked cigarettes”, “I have smoked cigarettes once or a few times (for fun or tried a puff)”, “I used to smoke cigarettes occasionally (not every day), but have quit now”, “I used to smoke cigarettes every day, but have quit now”, “I smoke cigarettes occasionally (not every day)” and “I smoke cigarettes every day”. EC use was assessed using a similar item [40].

Students were asked through which channel(s) they had been directly or indirectly exposed to messages promoting ECs during the past 30 days, with 11 options covering all the common sources and the options of “other channels” and “not exposed”. The sources were categorised into 7 types of advertising media [41]: 1) Broadcast media (“TV”, “Movies”); 2) Online media (“Video sites (e.g. YouTube)”, “Social/smartphone apps (e.g. Facebook, Weibo, Instagram, Twitter)”, and “Other websites”); 3) Print media (“Print materials”); 4) Outdoor media (“Billboard”); 5) Specialty media (“Materials with electronic cigarette brand logos”); 6) Point-of-sale marketing or physical objects (“Newspaper stands”, “Convenience stores”, and “Other stores or supermarkets”); 7) Other sources (“Other channels”). Exposure to advertising was converted into a dichotomous variable (not exposed and exposed) and an ordinal categorical variable (not exposed, exposed to 1 type, and exposed to ≥2 types).

We assessed two aspects of respondents’ attitudes towards EC use [42]. One question was “Do you think e-cigarette use will harm your health?” (options: “definitely not”, “probably not”, “probably will”, and “definitely will”); choosing any options other than “definitely will” indicated uncertainty that EC use harms health. Another question was “Your attitude towards e-cigarette use is” (options: “very negative”, “negative”, “neutral”, “positive” and “very positive”); choosing any options other than “negative” or “very negative” indicated tolerant attitudes towards EC use.

We also assessed two aspects of respondents’ attitudes towards the tobacco industry: “Do you think the tobacco companies are a respectable industry?” and “Do you think tobacco companies do everything they can to make young people smoke?” Both of these questions had options of “definitely not”, “probably not”, “probably yes” and “definitely yes” [39]. Choosing “probably yes” or “definitely yes” indicated a perception that the tobacco industry is respectable for the former question, and a belief that tobacco companies make youth smoke for the latter question.

We included two of the three items in the Pierce’s scale of susceptibility to smoking (copyright: freely available in the public domain), [43] which has been shown to predict smoking initiation and progression, [44] and we replaced cigarettes with ECs to assess adolescent susceptibility to EC use. The two items that we used were “Do you think you will use e-cigarettes in the next 12 months” and “Will you use e-cigarettes if one of your good friends offers you one?”, with the same options “definitely not”, “probably not”, “probably yes” and “definitely yes”. Students who chose “definitely not” for both items were deemed non-susceptible, and otherwise susceptible.

Students also reported their sex, age, grade, perceived family affluence (“relatively poor”, “poor to average”, “average”, “average to rich” and “relatively rich”), [45] and whether any of their parents used cigarettes or ECs.

### Statistical analyses

Proportions were weighted by sex, age and grade distribution of the target population provided by the Education Bureau of the Hong Kong SAR Government. Associations were assessed using generalised linear mixed models with a “logit” link function and random intercept accounting for school clustering effects with R (v3.5.1) package “lme4 (v1.1-21)”. Since age and grade are highly correlated, we only adjusted for grade together with sex, perceived family affluence and parental use of cigarettes and ECs. We examined associations between 1) exposure to EC advertising and attitudes towards ECs, 2) exposure to EC advertising and attitudes towards the tobacco industry, 3) attitudes towards ECs and susceptibility to EC use, 4) attitudes towards the tobacco industry and susceptibility to EC use, and 5) exposure to EC advertising and susceptibility to EC use through the potential mediation effects of attitudes towards ECs and the tobacco industry. Mediation effects were examined using multiple mediation analysis methods with R package “mma (v8.0–0)” [46]. The algorithms for the estimation of indirect effects were repeated 20 times with 50 times of bootstrap resampling. We did not consider random effects in the mediation analysis, as well-established multilevel statistical methods for assessing mediation with a binary outcome and multiple (≥ 3)  mediators are lacking [47].

## Results

Of the 8828 students who submitted a questionnaire, 1.4% were excluded because of extensive missing or inconsistent information. In the remaining 8704 students, 7082 who reported never using cigarettes or ECs were included in the analyses.

Table 1 shows that the mean age (standard deviation) was 14.9 (1.9) years, 50.8% were boys, and 36.2% reported parental cigarette/EC use. Over a quarter (28.8%) of students reported exposure to ≥1 type of EC advertising in the past 30 days, including 1 type (21.5%) and 2 or more types (7.3%). Broadcast media (TV and movies) was the most prevalent type (16.6%), followed by online media (7.6%) and point-of-sale marketing or physical objects (7.6%) (Supplementary Table 1). More than three-quarters (76.3%) of students had negative attitudes towards ECs, but less (69.1%) were certain that EC use harms health. As regards attitudes towards the tobacco industry, 74.4% of students did not regard tobacco companies as respectable but only 50.0% perceived that tobacco companies make youth smoke. Most (93.1%) students reported that they would definitely not use ECs in the next 12 months even if they were offered ECs by good friends; the rest (6.9%) were deemed susceptible to use ECs.

**Table 1:** Demographic characteristics and distributions of EC related exposure, attitudes and susceptibility

	N^a^	(%)^b^
**Sex (male)**	3821	50.8
**Age, years (SD)**^c^	14.8 (1.7)	14.9 (1.9)
**Grade**		
Secondary 1	1159	17.6
Secondary 2	1311	16.7
Secondary 3	1514	16.3
Secondary 4	1440	16.8
Secondary 5	1412	17.4
Secondary 6	246	15.2
**Perceived family affluence**		
Relatively poor	476	6.8
Poor to average	1753	25.8
Average	4068	56.9
Average to rich	659	9.0
Relatively rich	107	1.5
**Parental cigarette/EC use**	2534	36.2
**Number of types of EC advertising exposure**		
0	4888	71.2
1	1462	21.5
≥ 2	528	7.3
**EC use harms health**		
Definitely not	295	3.8
Probably not	337	4.8
Probably will	1631	22.4
Definitely will	4811	69.1
**Attitude towards EC use**		
Very negative	3318	47.8
Negative	2027	28.5
Neutral	1576	21.6
Positive	95	1.4
Very positive	54	0.7
**The tobacco industry is respectable**		
Definitely not	235	2.9
Probably not	1674	22.7
Probably yes	2677	38.3
Definitely yes	2475	36.1
**Tobacco companies make youth smoke**		
Definitely not	920	13.1
Probably not	2554	36.9
Probably yes	2457	34.7
Definitely yes	1135	15.3
**Being susceptible to EC use**	496	6.9

^a^Numbers unless otherwise stated

^b^Proportions unless otherwise stated. All proportions were weighted by age, sex, and grade distributions of the target population provided by the Education Bureau of the Hong Kong Government

^c^Mean age and standard deviation; unweighted under the column “N” and weighted under the column “%”

Table 2 shows that exposure to EC advertising in the past 30 days was associated with being uncertain that EC use harms health (AOR 1.17, 95% CI 1.05–1.31), being tolerant towards EC use (1.21, 1.07–1.36), and regarding the tobacco industry as respectable (1.42, 1.26–1.59); the magnitudes of these associations increased with the number of types of advertising exposed (0, 1, and ≥ 2) (all *p*-values < 0.01). However, EC advertising was not associated with perceiving that tobacco companies make youth smoke.

**Table 2:** Odds ratios of attitudes towards ECs and the tobacco industry by exposure to EC advertising

**a. Attitudes towards ECs**				
	**Uncertain that EC use harms health**		**Tolerant attitudes towards EC use**	
	**Crude OR**	**Adjusted OR**	**Crude OR**	**Adjusted OR**
	**(95% CI)**^a^	**(95% CI)**^b^	**(95% CI)**^a^	**(95% CI)**^b^
**Exposure to EC advertising**				
No	1	1	1	1
Yes	1.18 (1.05, 1.32)^**^	1.17 (1.05, 1.31)^**^	1.21 (1.07, 1.37)^**^	1.21 (1.07, 1.36)^**^
1 type	1.14 (1.00, 1.29)^*^	1.13 (1.00, 1.29)^*^	1.20 (1.05, 1.38)^**^	1.19 (1.04, 1.37)^*^
≥ 2 types	1.30 (1.08, 1.57)^**^	1.29 (1.06, 1.55)^**^	1.25 (1.02, 1.53)^*^	1.25 (1.02, 1.53)^*^
P for trend ^c^	< 0.01	< 0.01	< 0.01	< 0.01
**b. Attitudes towards the tobacco industry**				
	**The tobacco industry is respectable**		**Tobacco companies make youth smoke**	
	**Crude OR**	**Adjusted OR**	**Crude OR**	**Adjusted OR**
	**(95% CI)**^a^	**(95% CI)**^b^	**(95% CI)**^a^	**(95% CI)**^b^
**Exposure to EC advertising**				
No	1	1	1	1
Yes	1.42 (1.27, 1.60)^***^	1.42 (1.26, 1.59)^***^	1.09 (0.98, 1.22)	1.09 (0.98, 1.22)
1 type	1.36 (1.20, 1.55)^***^	1.36 (1.19, 1.55)^***^	1.08 (0.96, 1.22)	1.08 (0.96, 1.22)
≥ 2 types	1.59 (1.31, 1.92)^***^	1.58 (1.30, 1.91)^***^	1.12 (0.93, 1.34)	1.11 (0.92, 1.33)
P for trend ^c^	< 0.001	< 0.001	0.11	0.12

^*^P < 0.05, ^**^P < 0.01, ^***^P < 0.001

^a^CI = confidence interval. OR = odds ratio

^b^With adjustment for sex, grade, perceived family affluence, parental cigarette/EC use and school clustering effects

^c^*P* values for linear trends of exposure to 0, 1, and ≥ 2 types of EC advertising

Regression analyses were conducted to examine the associations of the four attitudinal variables with susceptibility to EC use, mutually adjusted for the attitudinal variables (Table 3). Being uncertain that EC use harms health (AOR 1.57, 95% CI 1.28–1.93), being tolerant towards ECs (3.30, 2.68–4.06), and regarding the tobacco industry as respectable (1.70, 1.39–2.07) were associated with being susceptible to EC use. However, perceiving that tobacco companies make youth smoke was not associated with susceptibility to EC use.

**Table 3:** Odds ratios of susceptibility to EC use by attitudes towards ECs and the tobacco industry

	Being susceptible to use ECs	
	Crude OR	Adjusted OR
	(95% CI)^a^	(95% CI)^b^
**a. Attitudes towards ECs**		
**EC use harms health**		
Certain	1	1
Uncertain	2.40 (2.00, 2.90)^***^	1.57 (1.28, 1.93)^***^
**Tolerant attitudes towards EC use**		
No	1	1
Yes	4.17 (3.45, 5.04)^***^	3.30 (2.68, 4.06)^***^
**b. Attitudes towards the tobacco industry**		
**The tobacco industry is respectable**		
No	1	1
Yes	2.13 (1.77, 2.58)^***^	1.70 (1.39, 2.07)^***^
**Tobacco companies make youth smoke**		
No	1	1
Yes	0.87 (0.72, 1.05)	1.01 (0.83, 1.23)

***P < 0.001

^a^CI = confidence interval. OR = odds ratio

^b^Adjusted for sex, grade, perceived family affluence, parental cigarette/EC use, other attitudinal variables in the table, and school clustering effects

**Fig. 1:**
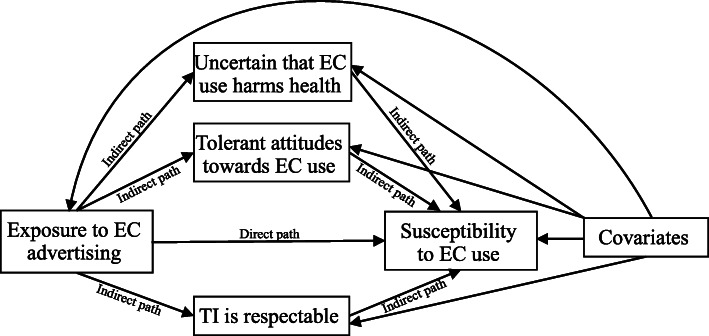
Mediation effects of perceptions of ECs and the tobacco industry between exposure to EC advertising and susceptibility to EC use. Notes: All associations were adjusted for the covariates, including sex, grade, perceived family affluence, and parental cigarette/EC use. Abbreviations: EC = e-cigarette, TI = tobacco industry

Mediation analysis (Fig. 1) was conducted between exposure to EC advertising and susceptibility to EC use, including the mediators of attitudes towards ECs and the tobacco industry, but not the perception that tobacco companies make youth smoke because it was not associated with exposure to EC advertising or susceptibility to EC use. Table 4 shows that exposure to EC advertising was associated with being susceptible to EC use with a total effect of 2.00 (AOR, 95% CI 1.56–2.48), and the mediation effects through all the three included mediators were statistically significant. The relative direct and indirect effects were calculated by dividing the respective effects by the total effect. Of the total effect, 8.2% (95% CI 3.4–14.3%) was mediated by tolerant attitudes towards ECs; less than 5% was mediated by uncertainty that EC use harms health (3.1%) and the perception that the tobacco industry is respectable (4.9%), with the joint effects of the three variables accounting for 16.3%. After taking all mediators into account, only 16.6% of the variation in susceptibility to EC use was explained by the indirect effects.

**Table 4:** Causal mediation analysis of attitudes between exposure to advertising and use susceptibility^a^

	Indirect effects	Relative effects %
	(95% CI)^b^	(95% CI)
**Mediation effects**		
Uncertain that EC use harms health	1.02 (1.01, 1.04)^***^	3.1 (0.7, 5.4)
Tolerant attitudes towards EC use	1.06 (1.03, 1.09)^***^	8.2 (3.4, 14.3)
The tobacco industry is respectable	1.03 (1.01, 1.06)^***^	4.9 (1.6, 8.6)
Joint effects ^c^	1.12 (1.07, 1.17)^***^	16.3 (9.5, 24.6)
**Direct effect**	1.78 (1.40, 2.20)^***^	83.4 (75.7, 90.1)
**Total effect**	2.00 (1.56, 2.48)^***^	–

^***^P < 0.001

^a^Not exposed to EC advertising = 0 and exposed to EC advertising = 1; not susceptible to EC use = 0 and susceptible to EC use = 1

^b^Adjusted odds ratio, with adjustment for sex, grade, perceived family affluence, parental cigarette/EC use and school clustering effects. CI = confidence interval

^c^Joint effects of the three mediators

## Discussion

The present results showed that exposure to EC advertising was associated with tolerant attitudes towards ECs and less perceived harm in adolescents who had never used ECs or cigarettes, which was consistent with previous research [31, 48, 49]; the magnitudes of these associations increased with the number of types of EC advertising exposed. Also, we found that exposure to EC advertising was associated with perceiving the tobacco industry as respectable. This concurred with previous studies showing that advertising could downplay the harm of EC use, made tobacco use more acceptable, and portrayed the industry as positive in non-smoking adolescents [9, 50]. However, students’ perceptions that tobacco companies make youth smoke was not associated with exposure to advertising or susceptibility to EC use. Despite these results, further studies are needed to investigate whether exposed adolescents realise they are targeted by EC companies.

Exposure to EC advertising were associated with susceptibility to trying ECs, [28, 29, 51, 52] and we further showed the AORs were similar when exposed to online and traditional broadcast media (Supplementary Table 1). Tobacco advertising in traditional media are increasingly banned globally. However, EC advertising surges in youth-oriented new media, and greatly extends its reach at low costs in recent years [11, 12, 27]. As tobacco use at young ages predicts regular use in adulthood and most established smokers initiate smoking in adolescence, [53–55] the online media become essential for recruiting young users of new tobacco products. More stringent regulations of tobacco advertising in online media are needed to curb adolescent use of new products.

A new finding from this research was that the association between exposure to EC advertising and susceptibility to EC use was only weakly mediated by tolerant attitudes towards ECs and the tobacco industry. Other studies have shown that adolescent EC use was largely unaffected by perceived health risks or social norms, but was related to curiosity, use by friends or family members, and availability of diverse flavours [56, 57]. Also, the characteristics of ECs that appealed to youth included fancy appearances, high-tech designs, kid-friendly flavours, “take your freedom back” (e.g. using ECs in no-smoking areas), and “back to school” (e.g. using ECs secretly at school) [56, 58]. These results suggested that restrictions on health claims, such as “harm modification” and “safer than cigarettes”, would be insufficient to prevent EC use in adolescents considering the minor mediation effects of harm perceptions.

The effects of EC advertising on adolescent use of ECs have not been reported in non-western countries or regions, and the present study can provide useful information for similar regions without relevant data. In Hong Kong, less than one third of adolescents (28.8%) were exposed to EC advertising in the past month and the prevalence of EC use was low (0.8% current use and 8.7% ever use in 2016/17) [24]. In contrast, most young people recalled past-month exposure in 2016/17 in Canada (74%), England (83%) and the US (81%) [59]. As large-scale EC marketing campaigns were absent in Hong Kong, students were more likely to be exposed to low-intensity advertising such as scenes of EC use in broadcast media and displays at point-of-sale. EC promotion in social media was also sporadic and non-official, with around 20–40 local channels (most followers were youths), groups or profiles in YouTube, Facebook or Instagram, and the common forms were promotional videos/images and customer testimonials. Despite the lower intensity of exposure to EC advertising, its associations with attitudes and susceptibility were comparable to those in Western countries, [29, 48, 51] which calls for prompt and stringent regulations on EC advertising even in low-prevalence regions.

However, due to the cross-sectional design, we could not determine the temporal sequence of exposure to EC advertising, attitudes and susceptibility to EC use. Susceptible respondents may have better recall of their exposure to EC advertising. Also, the large remaining direct effects might have been due to unmeasured confounders and/or mediators. For example, students spending more time on social media might have had higher risks of being exposed to EC advertising and susceptible to EC use. However, these limitations may also exist in cohort studies. As randomised trials of EC advertising would be unethical, ecological momentary assessments can be used in future studies to clarify temporality [60]. Finally, we asked adolescents to report exposures to messages promoting ECs, but respondents might have had different understandings of EC promotion. Other observational studies have assessed respondents’ recall of several well-known advertisements or visits to stores that typically contain these advertisements, [61, 62] but these are absent in Hong Kong. As children’s recognition of advertising approximates that of adults by ages 10–12, [63] we used the direct measurement of exposure through various channels in order to improve sensitivity, i.e. to avoid classifying exposed individuals as unexposed.

## Conclusions

Less than one third of secondary school students who had never used cigarettes or ECs were recently exposed to EC advertising in Hong Kong. Such exposure was associated with tolerant attitudes towards ECs and the tobacco industry, which was in turn associated with use susceptibility. The association between exposure to EC advertising and use susceptibility was only weakly mediated by attitudinal factors. The perception that tobacco companies make youth smoke was not associated with exposure to EC advertising or susceptibility to EC use.

This study raises an alarm to other low-prevalence regions that even exposure to low-intensity EC advertising could influence adolescents’ use susceptibility, in which attitudinal factors played a weak mediation role. Regulations of health claims in EC advertising would be insufficient to protect youth, and the ban on tobacco advertising and promotion, including that of EC products, should be enforced in both traditional and new media.

## Supplementary information

**Additional file 1: Supplementary Table 1.** Prevalence of exposure to various types of EC advertising in the past 30 days and the associations with susceptibility to EC use^a^.

## Data Availability

The raw data are confidential and cannot be made freely accessible. Dataset can be requested with appropriate reasons and permission from The Food and Health Bureau, the Government of Hong Kong Special Administrative Region. For requests, please contact the corresponding author.

## Abbreviations

EC: E-cigarette
SD: Standard deviation
AOR: Adjusted odds ratio
CI: Confidence interval
TI: Tobacco industry
