# Supplementary material for: Exposure to e-cigarette advertising, attitudes, and use susceptibility in adolescents who had never used e-cigarettes or cigarettes
Source: BMC Public Health. 2020 Sep 4;20:1349. doi: 10.1186/s12889-020-09422-w (PMC7650221; doi:10.1186/s12889-020-09422-w)
Supplement: Supplementary file 1 — Additional file 1: Supplementary Table 1. Prevalence of exposure to various types of EC advertising in the past 30 days and the associations with susceptibility to EC usea. [file 12889_2020_9422_MOESM1_ESM.docx]

| Supplementary Table 1. Prevalence of exposure to various types of EC advertising in the past 30 days and the associations with susceptibility to EC use^a^ | | | |
| --- | --- | --- | --- |
| **Types of EC advertising** | **N** | **%^b^** | **Adjusted OR** |
|  |  |  | **(95% CI)^c^** |
| Broadcast media | 1175 | 16.6 | 1.87 (1.50, 2.33)^***^ |
| Online Media | 514 | 7.6 | 1.53 (1.12, 2.09)^**^ |
| Print Media | 130 | 1.8 | 2.97 (1.86, 4.74)^***^ |
| Outdoor Media | 232 | 3.3 | 1.81 (1.20, 2.72)^**^ |
| Specialty Media | 116 | 1.8 | 1.56 (0.85, 2.87) |
| Point-of-sale marketing or physical objects | 568 | 7.6 | 1.00 (0.72, 1.40) |
| Other sources | 143 | 2.2 | 2.26 (1.39, 3.68)^**^ |
| ^*^P<0.05, ^**^P<0.01, ^***^P<0.001. | | | |
| ^a^ Not exposed to the corresponding type = 0 and exposed to the corresponding type = 1; non-susceptibility to EC use = 0 and having susceptibility to EC use = 1. | | | |
| ^b^ Proportions were weighted by age, sex, and grade distributions of the target population provided by the Education Bureau of the Hong Kong Government. | | | |
| ^c^ Odds ratio, with adjustment for sex, grade, perceived family affluence, parental cigarette/EC use and school clustering effects. CI=confidence interval. | | | |
